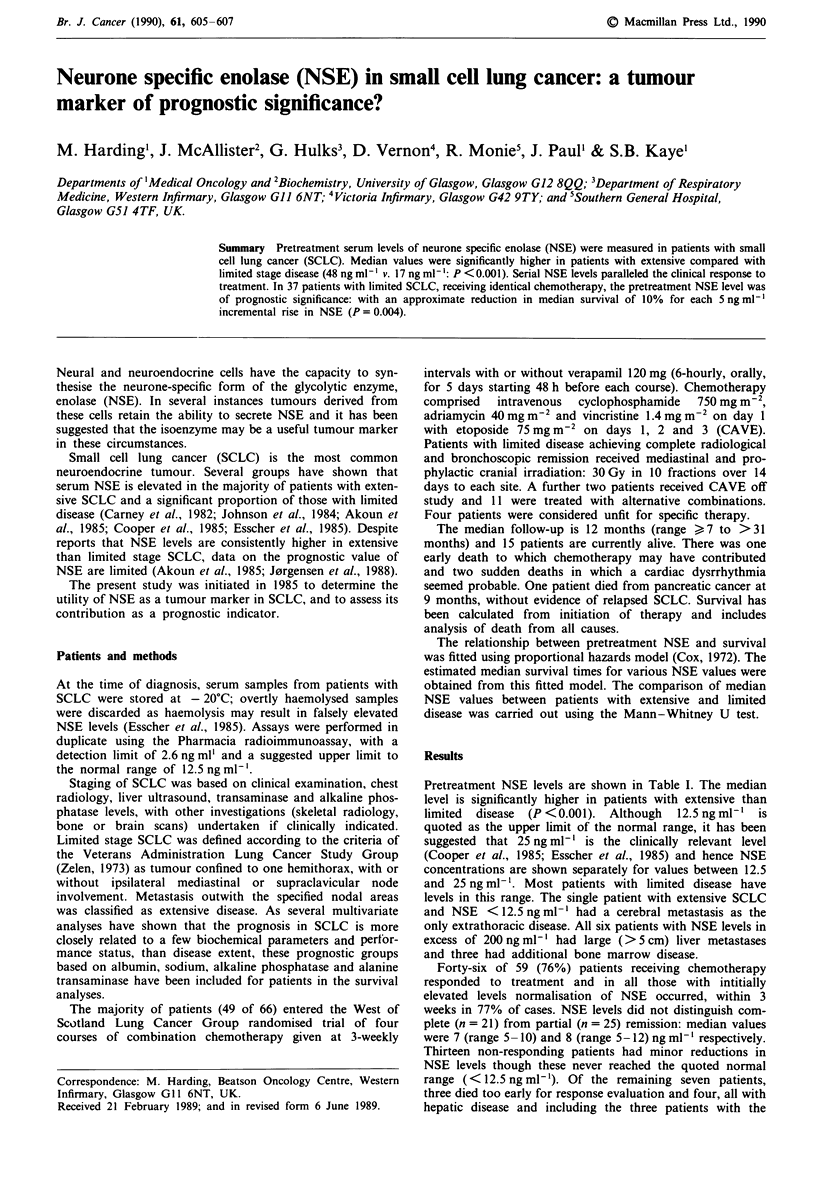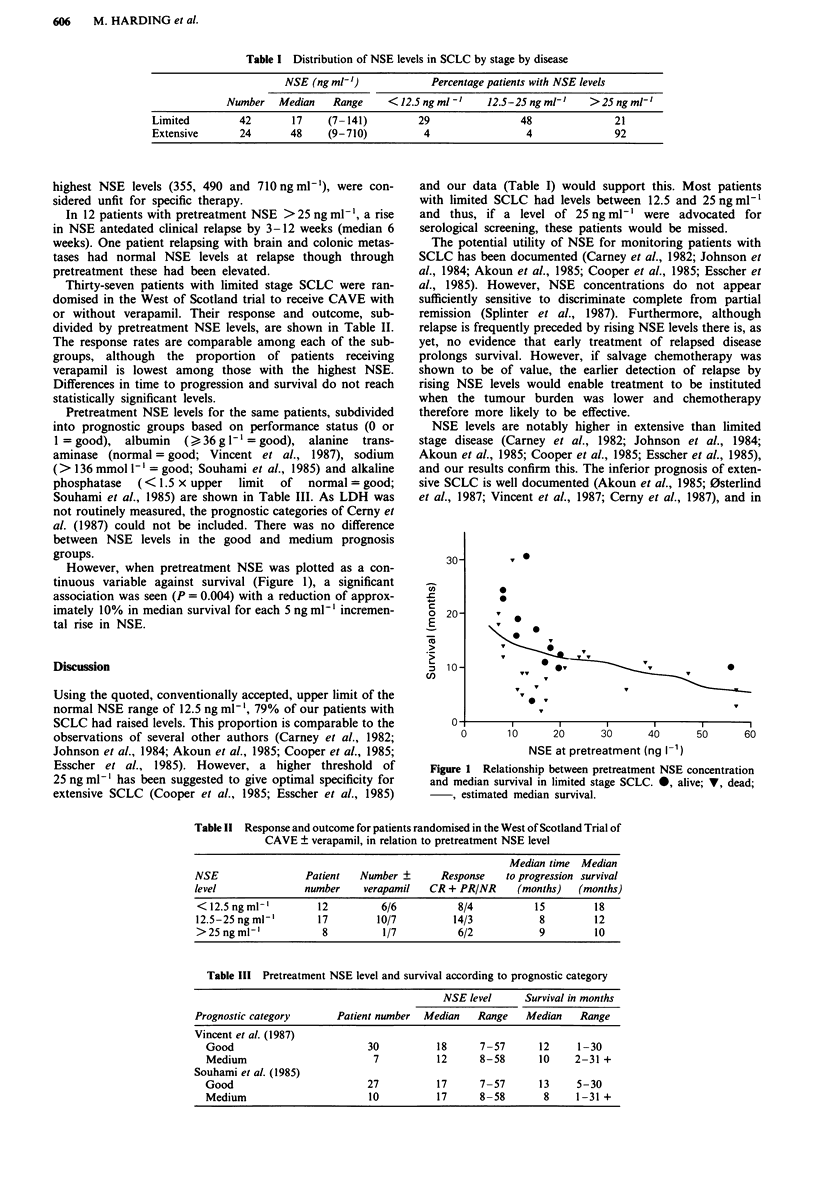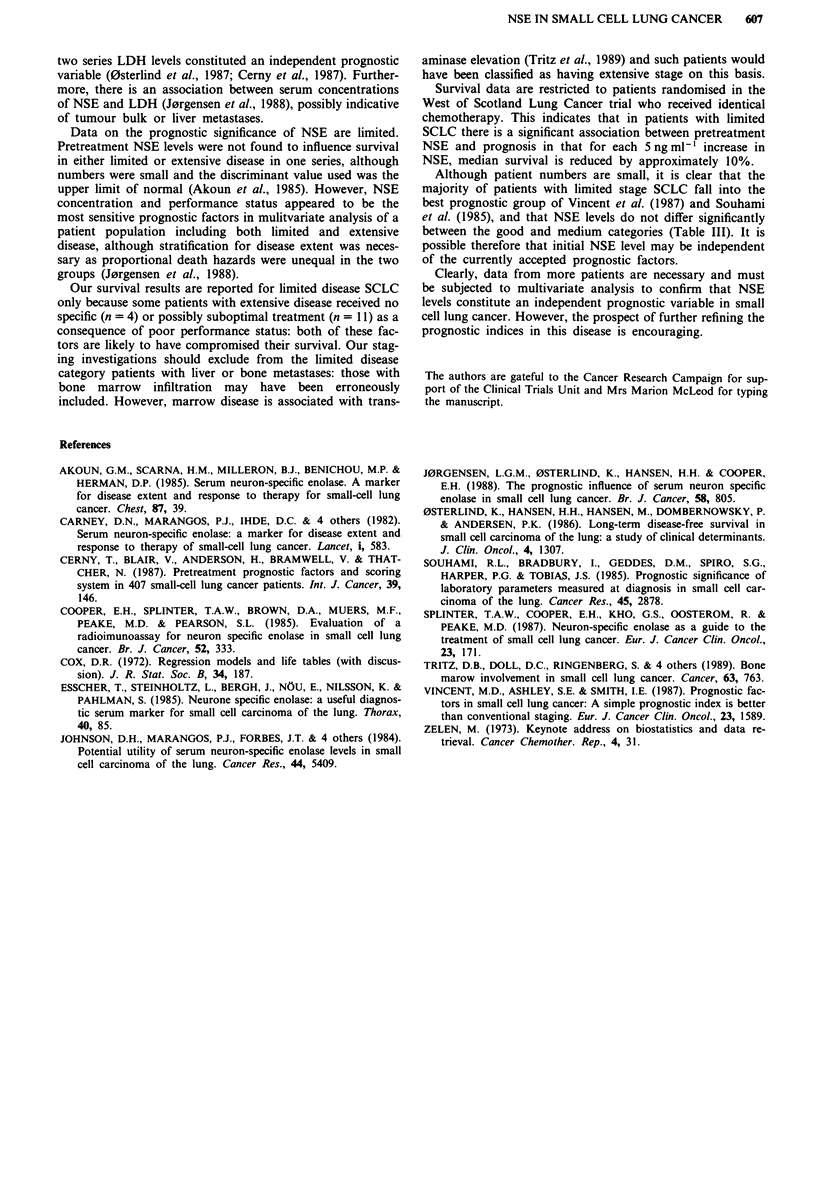# Neurone specific enolase (NSE) in small cell lung cancer: a tumour marker of prognostic significance?

**DOI:** 10.1038/bjc.1990.134

**Published:** 1990-04

**Authors:** M. Harding, J. McAllister, G. Hulks, D. Vernon, R. Monie, J. Paul, S. B. Kaye

**Affiliations:** Department of Medical Oncology, University of Glasgow, UK.

## Abstract

Pretreatment serum levels of neurone specific enolase (NSE) were measured in patients with small cell lung cancer (SCLC). Median values were significantly higher in patients with extensive compared with limited stage disease (48 ng ml-1 v. 17 ng ml-1: P less than 0.001). Serial NSE levels paralleled the clinical response to treatment. In 37 patients with limited SCLC, receiving identical chemotherapy, the pretreatment NSE level was of prognostic significance: with an approximate reduction in median survival of 10% for each 5 ng ml-1 incremental rise in NSE (P = 0.004).


Br. J. Cancer (1990), 61, 605-607 © Macmillan Press Ltd., 1990

### Neurone specific enolase (NSE) in small cell lung cancer: a tumour marker of prognostic significance?

M. Harding[1], J. McAllister[2], G. Hulks[3], D. Vernon[4], R. Monie[5], J. Paul[1] & S.B. Kaye[1]

*Departments of [1]Medical Oncology and [2]Biochemistry, University of Glasgow, Glasgow G12 8QQ; [3]Department of Respiratory Medicine, Western Infirmary, Glasgow G11 6NT; [4]Victoria Infirmary, Glasgow G42 9TY; and [5]Southern General Hospital, Glasgow G51 4TF, UK.*

**Summary** Pretreatment serum levels of neurone specific enolase (NSE) were measured in patients with small cell lung cancer (SCLC). Median values were significantly higher in patients with extensive compared with limited stage disease (48 ng ml$^{-1}$ *v.* 17 ng ml$^{-1}$: $P<0.001$). Serial NSE levels paralleled the clinical response to treatment. In 37 patients with limited SCLC, receiving identical chemotherapy, the pretreatment NSE level was of prognostic significance: with an approximate reduction in median survival of 10% for each 5 ng ml$^{-1}$ incremental rise in NSE ($P = 0.004$).

Neural and neuroendocrine cells have the capacity to synthesise the neurone-specific form of the glycolytic enzyme, enolase (NSE). In several instances tumours derived from these cells retain the ability to secrete NSE and it has been suggested that the isoenzyme may be a useful tumour marker in these circumstances.

Small cell lung cancer (SCLC) is the most common neuroendocrine tumour. Several groups have shown that serum NSE is elevated in the majority of patients with extensive SCLC and a significant proportion of those with limited disease (Carney *et al.*, 1982; Johnson *et al.*, 1984; Akoun *et al.*, 1985; Cooper *et al.*, 1985; Esscher *et al.*, 1985). Despite reports that NSE levels are consistently higher in extensive than limited stage SCLC, data on the prognostic value of NSE are limited (Akoun *et al.*, 1985; Jørgensen *et al.*, 1988).

The present study was initiated in 1985 to determine the utility of NSE as a tumour marker in SCLC, and to assess its contribution as a prognostic indicator.

### Patients and methods

At the time of diagnosis, serum samples from patients with SCLC were stored at −20°C; overtly haemolysed samples were discarded as haemolysis may result in falsely elevated NSE levels (Esscher *et al.*, 1985). Assays were performed in duplicate using the Pharmacia radioimmunoassay, with a detection limit of 2.6 ng ml$^{1}$ and a suggested upper limit to the normal range of 12.5 ng ml$^{-1}$.

Staging of SCLC was based on clinical examination, chest radiology, liver ultrasound, transaminase and alkaline phosphatase levels, with other investigations (skeletal radiology, bone or brain scans) undertaken if clinically indicated. Limited stage SCLC was defined according to the criteria of the Veterans Administration Lung Cancer Study Group (Zelen, 1973) as tumour confined to one hemithorax, with or without ipsilateral mediastinal or supraclavicular node involvement. Metastasis outwith the specified nodal areas was classified as extensive disease. As several multivariate analyses have shown that the prognosis in SCLC is more closely related to a few biochemical parameters and performance status, than disease extent, these prognostic groups based on albumin, sodium, alkaline phosphatase and alanine transaminase have been included for patients in the survival analyses.

The majority of patients (49 of 66) entered the West of Scotland Lung Cancer Group randomised trial of four courses of combination chemotherapy given at 3-weekly intervals with or without verapamil 120 mg (6-hourly, orally, for 5 days starting 48 h before each course). Chemotherapy comprised intravenous cyclophosphamide 750 mg m$^{-2}$, adriamycin 40 mg m$^{-2}$ and vincristine 1.4 mg m$^{-2}$ on day 1 with etoposide 75 mg m$^{-2}$ on days 1, 2 and 3 (CAVE). Patients with limited disease achieving complete radiological and bronchoscopic remission received mediastinal and prophylactic cranial irradiation: 30 Gy in 10 fractions over 14 days to each site. A further two patients received CAVE off study and 11 were treated with alternative combinations. Four patients were considered unfit for specific therapy.

The median follow-up is 12 months (range $\geq 7$ to $>31$ months) and 15 patients are currently alive. There was one early death to which chemotherapy may have contributed and two sudden deaths in which a cardiac dysrrhythmia seemed probable. One patient died from pancreatic cancer at 9 months, without evidence of relapsed SCLC. Survival has been calculated from initiation of therapy and includes analysis of death from all causes.

The relationship between pretreatment NSE and survival was fitted using proportional hazards model (Cox, 1972). The estimated median survival times for various NSE values were obtained from this fitted model. The comparison of median NSE values between patients with extensive and limited disease was carried out using the Mann–Whitney U test.

### Results

Pretreatment NSE levels are shown in Table I. The median level is significantly higher in patients with extensive than limited disease ($P<0.001$). Although 12.5 ng ml$^{-1}$ is quoted as the upper limit of the normal range, it has been suggested that 25 ng ml$^{-1}$ is the clinically relevant level (Cooper *et al.*, 1985; Esscher *et al.*, 1985) and hence NSE concentrations are shown separately for values between 12.5 and 25 ng ml$^{-1}$. Most patients with limited disease have levels in this range. The single patient with extensive SCLC and NSE $<12.5$ ng ml$^{-1}$ had a cerebral metastasis as the only extrathoracic disease. All six patients with NSE levels in excess of 200 ng ml$^{-1}$ had large ($>5$ cm) liver metastases and three had additional bone marrow disease.

Forty-six of 59 (76%) patients receiving chemotherapy responded to treatment and in all those with intitially elevated levels normalisation of NSE occurred, within 3 weeks in 77% of cases. NSE levels did not distinguish complete ($n = 21$) from partial ($n = 25$) remission: median values were 7 (range 5–10) and 8 (range 5–12) ng ml$^{-1}$ respectively. Thirteen non-responding patients had minor reductions in NSE levels though these never reached the quoted normal range ($<12.5$ ng ml$^{-1}$). Of the remaining seven patients, three died too early for response evaluation and four, all with hepatic disease and including the three patients with the

Correspondence: M. Harding, Beatson Oncology Centre, Western Infirmary, Glasgow G11 6NT, UK.
Received 21 February 1989; and in revised form 6 June 1989.

**Table I** Distribution of NSE levels in SCLC by stage by disease

| | | NSE (ng ml$^{-1}$) | | Percentage patients with NSE levels | | |
|---|---|---|---|---|---|---|
| | Number | Median | Range | <12.5 ng ml$^{-1}$ | 12.5–25 ng ml$^{-1}$ | >25 ng ml$^{-1}$ |
| Limited | 42 | 17 | (7–141) | 29 | 48 | 21 |
| Extensive | 24 | 48 | (9–710) | 4 | 4 | 92 |

highest NSE levels (355, 490 and 710 ng ml$^{-1}$), were considered unfit for specific therapy.

In 12 patients with pretreatment NSE >25 ng ml$^{-1}$, a rise in NSE antedated clinical relapse by 3–12 weeks (median 6 weeks). One patient relapsing with brain and colonic metastases had normal NSE levels at relapse though through pretreatment these had been elevated.

Thirty-seven patients with limited stage SCLC were randomised in the West of Scotland trial to receive CAVE with or without verapamil. Their response and outcome, subdivided by pretreatment NSE levels, are shown in Table II. The response rates are comparable among each of the subgroups, although the proportion of patients receiving verapamil is lowest among those with the highest NSE. Differences in time to progression and survival do not reach statistically significant levels.

Pretreatment NSE levels for the same patients, subdivided into prognostic groups based on performance status (0 or 1 = good), albumin ($\geqslant$36 g l$^{-1}$ = good), alanine transaminase (normal = good; Vincent *et al.*, 1987), sodium (>136 mmol l$^{-1}$ = good; Souhami *et al.*, 1985) and alkaline phosphatase (<1.5 × upper limit of normal = good; Souhami *et al.*, 1985) are shown in Table III. As LDH was not routinely measured, the prognostic categories of Cerny *et al.* (1987) could not be included. There was no difference between NSE levels in the good and medium prognosis groups.

However, when pretreatment NSE was plotted as a continuous variable against survival (Figure 1), a significant association was seen ($P = 0.004$) with a reduction of approximately 10% in median survival for each 5 ng ml$^{-1}$ incremental rise in NSE.

### Discussion

Using the quoted, conventionally accepted, upper limit of the normal NSE range of 12.5 ng ml$^{-1}$, 79% of our patients with SCLC had raised levels. This proportion is comparable to the observations of several other authors (Carney *et al.*, 1982; Johnson *et al.*, 1984; Akoun *et al.*, 1985; Cooper *et al.*, 1985; Esscher *et al.*, 1985). However, a higher threshold of 25 ng ml$^{-1}$ has been suggested to give optimal specificity for extensive SCLC (Cooper *et al.*, 1985; Esscher *et al.*, 1985) and our data (Table I) would support this. Most patients with limited SCLC had levels between 12.5 and 25 ng ml$^{-1}$ and thus, if a level of 25 ng ml$^{-1}$ were advocated for serological screening, these patients would be missed.

The potential utility of NSE for monitoring patients with SCLC has been documented (Carney *et al.*, 1982; Johnson *et al.*, 1984; Akoun *et al.*, 1985; Cooper *et al.*, 1985; Esscher *et al.*, 1985). However, NSE concentrations do not appear sufficiently sensitive to discriminate complete from partial remission (Splinter *et al.*, 1987). Furthermore, although relapse is frequently preceded by rising NSE levels there is, as yet, no evidence that early treatment of relapsed disease prolongs survival. However, if salvage chemotherapy was shown to be of value, the earlier detection of relapse by rising NSE levels would enable treatment to be instituted when the tumour burden was lower and chemotherapy therefore more likely to be effective.

NSE levels are notably higher in extensive than limited stage disease (Carney *et al.*, 1982; Johnson *et al.*, 1984; Akoun *et al.*, 1985; Cooper *et al.*, 1985; Esscher *et al.*, 1985), and our results confirm this. The inferior prognosis of extensive SCLC is well documented (Akoun *et al.*, 1985; Østerlind *et al.*, 1987; Vincent *et al.*, 1987; Cerny *et al.*, 1987), and in

**Figure 1** Relationship between pretreatment NSE concentration and median survival in limited stage SCLC. ●, alive; ▼, dead; ——, estimated median survival.

**Table II** Response and outcome for patients randomised in the West of Scotland Trial of CAVE ± verapamil, in relation to pretreatment NSE level

| NSE level | Patient number | Number ± verapamil | Response CR + PR/NR | Median time to progression (months) | Median survival (months) |
|---|---|---|---|---|---|
| <12.5 ng ml$^{-1}$ | 12 | 6/6 | 8/4 | 15 | 18 |
| 12.5–25 ng ml$^{-1}$ | 17 | 10/7 | 14/3 | 8 | 12 |
| >25 ng ml$^{-1}$ | 8 | 1/7 | 6/2 | 9 | 10 |

**Table III** Pretreatment NSE level and survival according to prognostic category

| | | NSE level | | Survival in months | |
|---|---|---|---|---|---|
| Prognostic category | Patient number | Median | Range | Median | Range |
| Vincent *et al.* (1987) | | | | | |
| Good | 30 | 18 | 7–57 | 12 | 1–30 |
| Medium | 7 | 12 | 8–58 | 10 | 2–31 + |
| Souhami *et al.* (1985) | | | | | |
| Good | 27 | 17 | 7–57 | 13 | 5–30 |
| Medium | 10 | 17 | 8–58 | 8 | 1–31 + |

two series LDH levels constituted an independent prognostic variable (Østerlind *et al.*, 1987; Cerny *et al.*, 1987). Furthermore, there is an association between serum concentrations of NSE and LDH (Jørgensen *et al.*, 1988), possibly indicative of tumour bulk or liver metastases.

Data on the prognostic significance of NSE are limited. Pretreatment NSE levels were not found to influence survival in either limited or extensive disease in one series, although numbers were small and the discriminant value used was the upper limit of normal (Akoun *et al.*, 1985). However, NSE concentration and performance status appeared to be the most sensitive prognostic factors in mulitvariate analysis of a patient population including both limited and extensive disease, although stratification for disease extent was necessary as proportional death hazards were unequal in the two groups (Jørgensen *et al.*, 1988).

Our survival results are reported for limited disease SCLC only because some patients with extensive disease received no specific ($n = 4$) or possibly suboptimal treatment ($n = 11$) as a consequence of poor performance status: both of these factors are likely to have compromised their survival. Our staging investigations should exclude from the limited disease category patients with liver or bone metastases: those with bone marrow infiltration may have been erroneously included. However, marrow disease is associated with transaminase elevation (Tritz *et al.*, 1989) and such patients would have been classified as having extensive stage on this basis.

Survival data are restricted to patients randomised in the West of Scotland Lung Cancer trial who received identical chemotherapy. This indicates that in patients with limited SCLC there is a significant association between pretreatment NSE and prognosis in that for each 5 ng ml$^{-1}$ increase in NSE, median survival is reduced by approximately 10%.

Although patient numbers are small, it is clear that the majority of patients with limited stage SCLC fall into the best prognostic group of Vincent *et al.* (1987) and Souhami *et al.* (1985), and that NSE levels do not differ significantly between the good and medium categories (Table III). It is possible therefore that initial NSE level may be independent of the currently accepted prognostic factors.

Clearly, data from more patients are necessary and must be subjected to multivariate analysis to confirm that NSE levels constitute an independent prognostic variable in small cell lung cancer. However, the prospect of further refining the prognostic indices in this disease is encouraging.

The authors are gateful to the Cancer Research Campaign for support of the Clinical Trials Unit and Mrs Marion McLeod for typing the manuscript.

### References

AKOUN, G.M., SCARNA, H.M., MILLERON, B.J., BENICHOU, M.P. & HERMAN, D.P. (1985). Serum neuron-specific enolase. A marker for disease extent and response to therapy for small-cell lung cancer. *Chest*, **87**, 39.

CARNEY, D.N., MARANGOS, P.J., IHDE, D.C. & 4 others (1982). Serum neuron-specific enolase: a marker for disease extent and response to therapy of small-cell lung cancer. *Lancet*, **i**, 583.

CERNY, T., BLAIR, V., ANDERSON, H., BRAMWELL, V. & THATCHER, N. (1987). Pretreatment prognostic factors and scoring system in 407 small-cell lung cancer patients. *Int. J. Cancer*, **39**, 146.

COOPER, E.H., SPLINTER, T.A.W., BROWN, D.A., MUERS, M.F., PEAKE, M.D. & PEARSON, S.L. (1985). Evaluation of a radioimunoassay for neuron specific enolase in small cell lung cancer. *Br. J. Cancer*, **52**, 333.

COX, D.R. (1972). Regression models and life tables (with discussion). *J. R. Stat. Soc. B*, **34**, 187.

ESSCHER, T., STEINHOLTZ, L., BERGH, J., NÖU, E., NILSSON, K. & PAHLMAN, S. (1985). Neurone specific enolase: a useful diagnostic serum marker for small cell carcinoma of the lung. *Thorax*, **40**, 85.

JOHNSON, D.H., MARANGOS, P.J., FORBES, J.T. & 4 others (1984). Potential utility of serum neuron-specific enolase levels in small cell carcinoma of the lung. *Cancer Res.*, **44**, 5409.

JØRGENSEN, L.G.M., ØSTERLIND, K., HANSEN, H.H. & COOPER, E.H. (1988). The prognostic influence of serum neuron specific enolase in small cell lung cancer. *Br. J. Cancer*, **58**, 805.

ØSTERLIND, K., HANSEN, H.H., HANSEN, M., DOMBERNOWSKY, P. & ANDERSEN, P.K. (1986). Long-term disease-free survival in small cell carcinoma of the lung: a study of clinical determinants. *J. Clin. Oncol.*, **4**, 1307.

SOUHAMI, R.L., BRADBURY, I., GEDDES, D.M., SPIRO, S.G., HARPER, P.G. & TOBIAS, J.S. (1985). Prognostic significance of laboratory parameters measured at diagnosis in small cell carcinoma of the lung. *Cancer Res.*, **45**, 2878.

SPLINTER, T.A.W., COOPER, E.H., KHO, G.S., OOSTEROM, R. & PEAKE, M.D. (1987). Neuron-specific enolase as a guide to the treatment of small cell lung cancer. *Eur. J. Cancer Clin. Oncol.*, **23**, 171.

TRITZ, D.B., DOLL, D.C., RINGENBERG, S. & 4 others (1989). Bone marow involvement in small cell lung cancer. *Cancer*, **63**, 763.

VINCENT, M.D., ASHLEY, S.E. & SMITH, I.E. (1987). Prognostic factors in small cell lung cancer: A simple prognostic index is better than conventional staging. *Eur. J. Cancer Clin. Oncol.*, **23**, 1589.

ZELEN, M. (1973). Keynote address on biostatistics and data retrieval. *Cancer Chemother. Rep.*, **4**, 31.